# Editorial: New Insights Into Salinity Sensing, Signaling and Adaptation in Plants

**DOI:** 10.3389/fpls.2020.604139

**Published:** 2021-03-01

**Authors:** Honghong Wu, Camilla Beate Hill, Giovanni Stefano, Jayakumar Bose

**Affiliations:** ^1^Ministry of Agriculture Key Laboratory of Crop Ecophysiology and Farming System in the Middle Reaches of the Yangtze River, College of Plant Science & Technology, Huazhong Agricultural University, Wuhan, China; ^2^College of Agronomy and Biotechnology, China Agricultural University, Beijing, China; ^3^Western Crop Genetics Alliance, Agricultural Sciences, College of Science, Health, Engineering and Education, Murdoch University, Perth, WA, Australia; ^4^Department of Plant Biology, Michigan State University, East Lansing, MI, United States; ^5^Australian Research Council Centre of Excellence in Plant Energy Biology, School of Agriculture, Food and Wine, University of Adelaide, Adelaide, SA, Australia

**Keywords:** salinity stress response, mechanisms, ion homeostasis, metabolism, transcription factors, energy cost

## Energy Metabolism in Plants Under Salinity Stress

Plants under salt stress require additional energy supply to fuel salt tolerance mechanisms and growth. Bandehagh and Taylor establish that plants must strike a balance between energy supply and demand to maintain growth and development during salt stress. This review (1) summaries how salt stress affects different physiological and biochemical processes altering the abundance of different metabolites that are feeding into regular and alternative respiratory pathways and shunts; (2) critically analyses how these metabolic adjustments might help plants to tolerate the salt better; (3) identifies research gaps; and (4) proposes suggestions for future breeding programs targeting high energy-use efficiency. Farhat et al. studied oxidative phosphorylation of mitochondria by comparing mitochondria purified from the suspension cultures of a halophyte (*Cakile maritima*) and a closely related glycophyte (*Arabidopsis thaliana*) plant. The abundance of respiratory supercomplexes (monomeric complex I, dimeric complex III and I + III_2_ supercomplex) were found to be higher in halophyte mitochondria in comparison with glycophyte, implying the efficient electron transfer from complex I to complex III in halophyte mitochondria. Further, the stability of ATP synthase (complex V) was also found to be higher in the halophyte, suggesting that halophyte mitochondria are better equipped to supply the additional ATP required to support the salt stress response.

## Organic Compatible Solutes for Plant Salt Tolerance

The synthesis of organic compatible solutes is an important component for plant salt stress tolerance. In this regard, proline plays an important role in protecting plants from osmotic stress. Guan et al. show that overexpression of the genes from the D1-pyrroline-5-carboxylate synthetase enzyme, one of the key enzymes for proline synthesis, such as *PvP5CS1* and *PvP5CS2* increased salt tolerance in switchgrass. The relative expression levels of spermidine and spermine synthesis and metabolism-related genes were upregulated and downregulated in the *PvP5CS* overexpression (OE) transgenic plants and *PvP5CS* RNAi transformants, respectively. Their results also suggest that exogenously applied proline could accelerate polyamines metabolisms in salt-stressed switchgrass. Moreover, changes in lipid metabolism have previously been linked to responses to environmental stresses including salinity. In their study, Yu et al. investigated leaf and root lipidome profiles of two sweet potato (*Ipomoea batatas* L.) cultivars under salinity conditions and showed that salt tolerance is associated with changes in lipid metabolic processes. They also discovered the important role of phosphatidylserine (PS) in mediating enzyme activity, and exogenous application of PS alleviated the effects of NaCl tissue toxicity.

## Tissue Specific Salinity Tolerance

Synthesis of organic osmolytes for osmotic adjustment is desirable; however, *de nova* synthesis of these molecules requires a high energy cost, and it cannot be sustainable while excluding salt. Alternatively, plants could employ tissue tolerance mechanisms so that they can use Na^+^ and Cl^−^ for osmotic adjustment, saving energy instead of spending it on the synthesis of organic solutes. Using four rice varieties, Chakraborty et al. investigated the contribution of ionic discrimination and tissue tolerance in rice in terms of its energy cost. The results indicated that the two salt tolerance mechanisms, i.e., ionic selectivity and tissue tolerance, are distinct in rice. They also found that perhaps with a lower energy cost, unique rice varieties such as Kamini could effectively balance ionic discrimination vis-à-vis tissue tolerance to achieve salt tolerance. Furthermore, tissue specificity of employed salt tolerance mechanisms is known as an important factor which confers plant salinity stress tolerance. Liu, Shabala et al. investigated the differences in the operation of key ion transporters mediating ionic homeostasis in three rice varieties differing in salinity stress tolerance. The results showed that the superior K^+^ retention ability in both the mature and elongation zone of the rice root is the key trait conferring its differential salinity stress tolerance. They suggested that besides the superior ability to activate root H^+^-ATPase pump operation, this key trait is also related to the reduced sensitivity of K^+^ efflux channels to reactive oxygen species and the lower upregulation in *OsGORK* and higher upregulation of *OsAKT1*.

## Transporters Controlling Na^+^/K^+^ Ratio

A key trait which has long been recognized to improve salinity tolerance in many plants, is the maintenance of a low Na^+^/K^+^ ratio. Enhanced operation of salt overly sensitive 1 (*SOS1*; a Na^+^/H^+^ antiporter) has been shown to enable Na^+^ extrusion and thus confer salt tolerance in multiple plant species. Though SOS1 function at the root apex is well-established, how this transporter could function in the epidermis of the root mature zone and stele is unclear. Foster and Miklavcic employed biophysical modeling to show that SOS1 transport function is markedly different between the epidermis of the mature root zone and stele. In the epidermis, SOS1 restricts Na^+^ entry into the root cells reducing cytosolic Na^+^ levels of the root cells while in stele, SOS1 actively loads Na^+^ into the xylem, enhancing the flux of Na^+^ to the shoot. Besides SOS1 Na^+^/H^+^ antiporter, members of the HKT family proteins are well-known to protect plants against excess Na^+^ levels. Shohan et al. investigated the high-affinity K^+^ transporter HKT1;5 in different rice genotypes, including the salinity-sensitive *Oryza* genotype IR29, the salinity-tolerant *Oryza* genotype Pokkali, and the distantly related halophytic wild rice genotype *Porteresia coarctata*. A comparison of HKT1;5 sequences revealed four major amino acid substitutions, two of which were common for salinity-tolerant genotypes. Based on molecular modeling and structure validation, these two substitutions led to altered ion selectivity and uptake kinetics. These changes were hypothesized to allow salinity-tolerant genotypes to remove toxic Na^+^ and maintain the Na^+^/K^+^ ratio, improving survival under salinity stress conditions. Furthermore, Lu et al. cloned *CIPK9* gene (*Calcineurin B-like protein-interacting protein kinases*, CIPK) from the halophyte *Nitraria tangutorum* and characterized it in Arabidopsis plants. The Arabidopsis overexpression lines expressing *NtCIPK9* showed enhanced salt tolerance by regulating genes related to K^+^ nutrition. Moreover, maintaining Cl^−^ homeostasis is also important for plant salt tolerance. Wu and Li summarized the role of Cl^−^ transport in plant salt tolerance from the aspect of Cl^−^ transport, Cl^−^ exclusion, vacuolar Cl^−^ sequestration, the specificity of mechanisms employed in different plant species to control shoot Cl^−^ accumulation, and the identity of Cl^−^ transport channels and transporters. Authors argued that vacuolar Cl^−^ sequestration could play a role in salt tolerance if the plant species that have tonoplast potential amount to negative or positive tens of mV.

## Exogenous Elicitors, Hormones, and Chemicals for Plant Salinity Stress Adaption

Improving salt tolerance through exogenous application of biostimulants, plant hormones, or chemicals, e.g., silicon, in plants has long been explored as a management option. Here, in this Research Topic, authors showed such improvement of tolerance is linked with better maintained low Na^+^/K^+^ ratio in elicitor or hormones treated plants under salinity. Ursolic acid (UA, 3b-hydroxy-12-urs-12-en-28-oic acid), a natural pentacyclic triterpenoid carboxylic acid which is widely present in vegetables and fruits, is a biostimulant/elicitor with antioxidant, apoptotic, anticancer, and anti-inflammatory properties in animal models. Its role and underlying mechanisms in improving plant salinity tolerance are still ambiguous. Long et al. showed that UA application improved rice salt tolerance by enabling better ROS homeostasis, triggering nitric oxide (NO) production, and maintaining a lower cytosolic Na^+^/K^+^ ratio. The results suggest that both UA and NO are required together to develop a salt tolerance response in rice. Brassinolide is one of the plant hormones implicated in regulating plant growth, development and abiotic stress tolerance. Su et al. investigated how brassinolide application could alter the salt tolerance of apple (*Malus hupehensis* Rehd) and found that brassinolide can alleviate salt stress by promoting the activity of reactive oxygen species (ROS) scavenging enzymes and the production of soluble sugars and proline. Brassinolide application reduced Na^+^ accumulation and increased K^+^ content in shoots and roots under salt stress by regulating the expression levels of Na^+^(K^+^)/H^+^ antiporter genes. Moreover, the abscisic acid (ABA) signal is known to play an important role in reducing the stomatal opening to prevent water loss and wilting in plants under early periods of salt stress. Niu et al. found that the root-sourced ABA signal resulted in rapid stomatal closure and increased salt resistance in cucumber grafted onto pumpkin. Their results also showed that compared with self-grafting, cucumbers grafted onto pumpkin had increased sensitivity to ABA, showing that pumpkin root increases the sensitivity of the cucumber scion to ABA translocated from the root to the shoot. Silicon (Si) is known to mitigate salinity stress in plants. However, the exact mechanism by which Si improves salt tolerance is not known. Khan et al. reviewed the possible mechanisms underlying Si-associated stress tolerance in plants through modulating Na^+^ balance, water status, reactive oxygen species homeostasis, photosynthesis, phytohormone levels, and compatible solutes. The authors suggest that exogenous Si application improves plant salt tolerance by enhancing antioxidant enzyme activities or blocking Na^+^ uptake and translocation.

## Mediators Sensing Salinity Stress Signals

Non-selective cation channels are a diverse group of ion channels capable of indiscriminately transporting many essential and toxic cations into plant cells during salinity stress. The model plant *Arabidopsis thaliana* has a family of 20 glutamate receptor-like proteins (GLRs) that have been suggested to function as non-selective cation channels. Wang et al. characterized one of the GRL members, AtGLR3.7, and its role in Ca^2+^ homeostasis. Interestingly they have identified a phosphorylation site (Serine-860) and showed that mutating Ser-860 to alanine abolished the interaction between AtGLR3.7 and the 14-3-3ω protein. They further established that the primary root growth of the GLR3.7-S860A overexpression lines was less sensitive to salt stress than the corresponding wild-type. The 14-3-3 proteins are known to be mediators of salt stress signals and part of the stress response network in C3 plants. Since very little is known about the role of this protein family in C4 plants in response to salinity stress, Liu, Jiang et al. investigated 14-3-3 proteins (*Si*GRFs, *G*eneral *R*egulatory *F*actor) in foxtail millet (*Setaria italica*), a C4 genetic model plant for cereals. They identified eight different 14-3-3 proteins in the foxtail millet genome and performed *in silico* tissue-specific expression profiling at several development stages under stress conditions, showing that 14-3-3 proteins were responsive to salinity stress. In the presence of salt stress, overexpression of *SiGRF1* in *Arabidopsis thaliana* increased the expression of several key flowering time genes leading to early flowering when compared with the untransformed wild type.

## Transcription Factors Involved in Plant Salinity Stress Response

A plant's response to salinity stress at the molecular level is earlier than the abovementioned physiological response. Transcription factors regulate gene expression by recognizing and binding to the specific cis-elements in the promoters of the target genes and play an important role in the plant stress response. Gai et al. identified and characterized 60 bZIP (basic leucine zipper) transcription factors encoding genes from two pepper genomes and found that *CabZIP25* positively modulates salinity stress tolerance in pepper. Under salt stress, *CabZIP25*-silenced pepper showed lower chlorophyll content than the control plants. Additionally, overexpression of *CabZIP25* in *Arabidopsis* showed an increased germination rate, fresh weight, chlorophyll content, and root lengths under salt stress compared to the wild type plants. Wang et al. found that *MdBZR1* (BRASSINAZOLE RESISTANT1) and *MdBZR1-2 like* transcription factors could bind to the promoters of key GA (gibberellin acid) biosynthetic genes *MdGA20ox2* and *MdGA3ox1*, thus improving salt tolerance in apple by regulating gibberellin biosynthesis. Interestingly, Zhang et al. generated mutant lines of *zmcps-1* (ent-copalyl diphosphate synthase, one of the key enzymes for early steps of GA biosynthesis) and *zmcps-7* and found that this knockout mutation improved maize salt tolerance through enhancing vacuolar Na^+^ sequestration and maintaining ROS homeostasis. These results indicate that the regulatory role of GA in plant salt tolerance is complicated and more efforts are needed to study its role in plant stress responses. Unlike the positive regulatory role of *CabZIP25, MdBZR1*, and *MdBZR1-2like* in improving plant salt tolerance, Tang et al. showed that the ectopic overexpression of homeodomain-leucine zipper (HD-Zip) transcription factor *JcHDZ07* from physic nut (*Jatropha curcas* L.), one of the most promising bio energy supplying plants for tropical and subtropical regions, enhances salt sensitivity in Arabidopsis.

## Breeding for Salt Tolerance in Plants

In the past, attempts to develop salinity-tolerant plants have had limited success due to multigenicity of the trait as well as non-availability of suitable donors containing beneficial alleles for salinity tolerance. Rice is the most salt-sensitive cereal crop. Solis et al. discuss the potential challenges and opportunities of using wild rice species as a prime reservoir of genetic diversity for the improvement of cultivated rice. They summarize differences in salinity stress adaptation between cultivated and wild rice and propose several traits (such as tissue tolerance) as potential targets in future breeding programs. To date, over 127,000 cultivated and wild rice accessions have been reported, but only a few salt-tolerant cultivars harboring salt tolerance alleles have been identified. In this regard, a major QTL, *Saltol* comprising several salt-tolerant genes, was identified on the short arm of chromosome 1, accounting for more than 62% phenotypic variation under salinity in rice. Krishnamurthy et al. incorporated this *Saltol* into two high yielding rice varieties (Pusa44 and Sarjoo52) through marker-assisted backcross breeding and generated several near-isogenic lines with enhanced salt tolerance.

This Research Topic also contains research on salt tolerance mechanisms operating under mild salinity stress. Unlike salt-sensitive rice, some crops can grow under mild salinity stress. For example, sugar beet (*Beta vulgaris* L.) is a salt-tolerant crop, and the yield of sugar beet will not be affected up to 7.0 dS m^−1^ (**≈**67 mM NaCl). Furthermore, sweet sorghum can not only survive, but can increase its sugar content under saline conditions. Lv et al. summarize the physiological and molecular mechanisms involved in ion homeostasis, osmotic-adjustment, and reactive oxygen species scavenging that allow sugar beet to tolerate salt stress. Further, this review describes key candidate genes pivotal for sugar beet salt tolerance using ‘omics technologies’ and provides hints for genetic improvement and molecular breeding for crop salt tolerance. Yang et al. reviewed the physiological and biochemical responses of salt-stressed sweet sorghum and summarized that the major advantages of salt-tolerant sweet sorghum include: (1) improving Na^+^ exclusion ability to maintain root ion homeostasis to ensure a relatively low shoot Na^+^ concentration under saline conditions; (2) maintaining a high shoot sugar content under saline conditions which is enabled by protecting photosystems structures, enhancing photosynthetic performance and sucrose synthetase activity, and inhibiting sucrose degradation. Further, authors suggested that targeting the key genes related to the regulatory mechanisms could provide opportunities to breed more salt tolerant sweet sorghum.

Overall, we hope this Research Topic benefits plant breeders and land managers by delivering novel information and insights on the salinity stress response, signaling, and the adaptive mechanisms operating in plants.

## Author Contributions

All authors listed have made a substantial, direct and intellectual contribution to the work, and approved it for publication.

## Conflict of Interest

The authors declare that the research was conducted in the absence of any commercial or financial relationships that could be construed as a potential conflict of interest. The handling editor declared a past co-authorship with several of the authors, HW and JB.

